# Supplementary material for: Applying the advocacy coalition framework to wildlife management: Explaining policy change for damage mitigation in Japan
Source: PLoS One. 2025 Sep 12;20(9):e0331966. doi: 10.1371/journal.pone.0331966 (PMC12431195; doi:10.1371/journal.pone.0331966)
Supplement: S1 Table — (DOCX) [file pone.0331966.s001.docx]

S1 Table. The ACF hypotheses

| Coalition Hypothesis 1 (CH1) | On major controversies within a policy subsystem when policy core beliefs are in dispute, the lineup of allies and opponents tends to be rather stable over periods of a decade or so. |
| --- | --- |
| Coalition Hypothesis 2 (CH2) | Actors within an advocacy coalition will show substantial consensus on issues pertaining to the policy core, although less so on secondary aspects. |
| Coalition Hypothesis 3 (CH3) | Actors (or coalitions) will give up the secondary aspects of their belief systems before acknowledging weaknesses in the policy core. |
| Coalition Hypothesis 4 (CH4) | Within a coalition, administrative agencies will usually advocate more moderate positions than their interest group allies. |
| Coalition Hypothesis 5 (CH5) | Actors within purposive groups are more constrained in their expression of beliefs and policy positions than actors from material groups. |
| Learning Hypothesis1 (LH1) | Policy-oriented learning across belief systems is most likely when there is an intermediate level of informed conflict between two coalitions. This requires that (1) each has the technical resources to engage in such a debate, and (2) the conflict is between secondary aspects of one belief system and core elements of the other or, alternatively, between important secondary aspects of the two belief systems. |
| Learning Hypothesis2 (LH2) | Policy-oriented learning across belief systems is most likely when there exists a forum that is: (1) prestigious enough to force professionals from different coalitions to participate and (2) dominated by professional norms. |
| Learning Hypothesis 3 (LH3) | Problems for which accepted quantitative data and theory that exist are more conducive to policy-oriented learning across belief systems than those in which data and theory are generally qualitative, quite subjective, or altogether lacking. |
| Learning Hypothesis 4 (LH4) | Problems involving natural systems are more conducive to policy-oriented learning across belief systems than those involving purely social or political systems because in the former many of the critical variables are not themselves active strategists and because controlled experimentation is more feasible. |
| Learning Hypothesis 5 (LH5) | Even when the accumulation of technical information does not change the views of the opposing coalition, it can have important impacts on policy—at least in the short run—by altering the views of policy brokers. |
| Policy Change Hypothesis 1 (PCH1) | Significant perturbations external to the subsystem, a significant perturbation internal to the subsystem, policy-oriented learning, negotiated agreement, or some combination thereof is a necessary, but not sufficient, source of change in the policy core attributes of a governmental program. |
| Policy Change Hypothesis 2 (PCH2) | The policy core attributes of a governmental program in a specific jurisdiction will not be significantly revised as long as the subsystem advocacy coalition that instated the program remains in power within that jurisdiction—except when the change is imposed by a hierarchically superior jurisdiction. |

Source: Nohrstedt et al. (38)
