# Supplementary material for: Applying the advocacy coalition framework to wildlife management: Explaining policy change for damage mitigation in Japan
Source: PLoS One. 2025 Sep 12;20(9):e0331966. doi: 10.1371/journal.pone.0331966 (PMC12431195; doi:10.1371/journal.pone.0331966)
Supplement: S2 Table — (DOCX) [file pone.0331966.s002.docx]

S2 Table. Interview Guide

| Questions  1. Please tell me briefly about your career.  2. We would like to ask you about the deer and wild boar policy of the 1990s. Please answer in as much detail as possible.  ・What was your position at the time and what activities were you engaged in?  ・What policy proposals were you making for national and prefectural governments? (e.g., lifting the hunting ban on female deer to control the population) And what actions did you take to get your proposals accepted? (e.g., lobbying parliamentarians, holding study groups, establishing organizations, etc.)  ・Who were the people who supported or understood your proposals? (political parties, government officials, researchers, related organizations, etc.)  ・Who opposed your proposals? (Political parties, government officials, researchers, related organizations, etc.)  ・What do you think wildlife policy in the 1990s should have looked like? If you have any thoughts on the reasons why such policy ideas were not realized, please let us know.  ・Who were the people who were important in promoting or hindering changes in wildlife policy in the 1990s? Were there any scientific findings or ideas that promoted this?  ・Are there any other important phenomena that can help us understand the trends in wildlife policy in the 1990s? (e.g., decentralization) |
| --- |
| 3. Next, I would like to ask you about deer and wild boar policy in the 2000s. Please answer in as much detail as possible.  ・What was your position at the time and what activities were you involved in?  ・What policy proposals were you making for national and prefectural governments? And what actions did you take to get your proposals accepted?  ・Who were the people who supported or understood your proposals?  ・Who opposed your proposals?  ・What do you think wildlife policy in the 2000s should have looked like? If you have any thoughts on the reasons why these policy ideas were not realized, please let us know.  Who was important in promoting or hindering the changes in wildlife policy in the 2000s? Were there any scientific findings or ideas that promoted this?  ・Are there any other important phenomena that can help us understand the trends in wildlife policy in the 2000s? |
| 4.Next, I would like to ask you about deer and wild boar policy in the 2010s. Please answer in as much detail as possible.  ・What was your position at the time and what activities were you involved in?  ・What policy proposals were you making for national and prefectural governments? And what actions did you take to get your proposals accepted?  ・Who were the people who supported or understood your proposals?  ・Who opposed your proposals?  ・What do you think wildlife policy in the 2010s should have looked like? If you have any thoughts on the factors that prevented such policy ideas from being realized, please share them with us.  ・Who was important in promoting or hindering changes in wildlife policy in the 2010s? Were there any scientific findings or ideas that promoted this?  ・Are there any other important phenomena that can help us understand the trends in wildlife policy in the 2010s?  5. Finally, I would like to ask you about current and future deer and wild boar policy.  ・How do you evaluate Japan's current deer and wild boar policies?  ・What do you hope to see from the national government, prefectures, and municipalities in the future?  ・What issues do you think are important, and who do you think should take action to address them? Why?  ・Please tell me anything else you would like to add.  Thank you very much. |

Source: The Author
