# Supplementary material for: Applying the advocacy coalition framework to wildlife management: Explaining policy change for damage mitigation in Japan
Source: PLoS One. 2025 Sep 12;20(9):e0331966. doi: 10.1371/journal.pone.0331966 (PMC12431195; doi:10.1371/journal.pone.0331966)
Supplement: S3 Table — (DOCX) [file pone.0331966.s003.docx]

S3 Table. List of Organizations in policy subsystem

| **Attribute** | **Abbreviation** | **Organization name** | **Name in Japanese** | **number of interviewees^a^** |
| --- | --- | --- | --- | --- |
| Government | ME | The Ministry of Environment | 環境省 | 2 |
|  | MAFF | The Ministry of Agriculture, Forestry, Fisheries | 農林水産省 | 2 |
| Prefectural Government | Tochigi P | Tochigi Prefecture | 栃木県 | 0 |
|  | Shimane p | Shimane Prefecture | 島根県 | 0 |
|  | Shiga P | Shga Prefecture | 滋賀県 | 0 |
|  | Nagano P | Nagano Prefecture | 長野県 | 0 |
|  | Hyogo P | Hyogo Prefecture | 兵庫県 | 0 |
| Political Party (Right-Wing) | LDP | Liberal Democratic Party | 自民党 | 00 |
|  | Ishin P | Ishin Party | 日本維新の会 | 0 |
| Political Party (Center) | KP | Koumei Party | 公明党 | 0 |
| Political Party (Left-Wing) | DPJ | Democratic Party of Japan | 民主党 | 0 |
|  | SDP | Social Democratic Party | 社民党 | 0 |
|  | JCP | Japanese Communist Party | 日本共産党 | 0 |
| Hunting Group^b^ | JHA | Japan Hunting Association | 大日本猟友会 | 1 |
| Nature Conservation Group・Animal Protection Group | WWF-J | WWF-Japan | WWFジャパン | 0 |
|  | NACS-J | The Nature Conservation Society of Japan | 日本自然保護協会 | 1 |
|  | ALIVE | All Life In a Viable Environment | ALIVE | 0 |
|  | WPAN | Wildlife Protection Act Network | 鳥獣保護法改正を考えるネットワーク（野生生物保護法の制定をめざす全国ネットワーク） | 1 |
|  | JBFS | Japan Bear & Forest Society | 日本熊森協会 | 2 |
|  | JWCS | Japan Wildlife Conservation Society | 野生動物保全論研究会 | 0 |
| Research Institute | FFPRI | Forestry and Forest Products Research Institute | 森林総合研究所 | 2 |
|  | HERC | Hokkaido environmental Research center | 北海道環境科学研究センター | 1 |
|  | HPU | Hyogo Prefectural University | 兵庫県立大学 | 0 |
|  | UU | Utsunomiya University | 宇都宮大学 | 0 |
|  | TUAT | Tokyo University of Agriculture and Technology | 東京農工大学 | 1 |

Source: The Author

^a^ The number of interviewees listed in this table differs from the total number reported in the main text. This is because the table includes only actors who were part of the policy subsystem. In addition to these, interviews were also conducted with individuals who were not directly involved in the policy subsystem but were regularly engaged in the policy process, such as members of environmental consulting firms.

^b^ In Japan, the organizational structure of hunting associations consists of local hunting clubs that operate at the municipal level, prefectural hunting clubs that coordinate activities at the prefectural level, and the Japan Hunters’ Association (JHA), which oversees the entire national framework. While there are other hunter organizations besides JHA, they are small in scale and lack the political resources to influence national policymaking. Among these, only the JHA has the institutional capacity to exert influence on national wildlife policy. As such, the JHA is the sole hunter organization that participates in national-level policy discussions as the representative of hunters across Japan. Therefore, while multiple prefectural governments are listed in Table S3 due to their influence on national policymaking, only one hunter organization (JHA) is included, reflecting this institutional reality rather than any sampling bias. Based on the reviewer’s suggestion, this explanation has been added to clarify the context.
