# Supplementary material for: Applying the advocacy coalition framework to wildlife management: Explaining policy change for damage mitigation in Japan
Source: PLoS One. 2025 Sep 12;20(9):e0331966. doi: 10.1371/journal.pone.0331966 (PMC12431195; doi:10.1371/journal.pone.0331966)
Supplement: S4 Table — (DOCX) [file pone.0331966.s004.docx]

S4 Table. Coding Scheme

| **Fundamental normative precepts** |
| --- |
| **Orientation on basic value priorities.**  Hunting should be promoted to reduce damage caused by ungulates.：agree/disagree  Hunting should be regulated to protect ungulates.：agree/disagree |
| **Identification of groups or other entities whose welfare is of greatest concern.**  The welfare of wildlife damage victims should be given priority.：agree/disagree  The lives of wildlife should be prioritized.：agree/disagree  The welfare of hunters should be given priority.：agree/disagree |
| **Precepts with a substantial empirical component** |
| **Proper distribution of authority among levels of government.**  The central government should be responsible for wildlife management.：agree/disagree  Decentralization to prefectures should be encouraged.：agree/disagree  Decentralization to municipalities should be encouraged.：agree/disagree |
| **Secondary Aspects** |
| 1999 WPHA revision for ungulate management ：agree/disagree  2003 WPHA revision for ungulate management ：agree/disagree  2006 WPHA revision for ungulate management ：agree/disagree  2008 enactment of the ASMPDRAFFCW for ungulate management ：agree/disagree  2014 WPHA revision for ungulate management ：agree/disagree  Lifting of the ban on hunting female deer：agree/disagree  Involvement of the Self-Defense Forces in hunting activities：agree/disagree  Relaxation of regulations on trap hunting：agree/disagree  Public subsidies for hunting：agree/disagree |

Source: The Author
