# Supplementary material for: Applying the advocacy coalition framework to wildlife management: Explaining policy change for damage mitigation in Japan
Source: PLoS One. 2025 Sep 12;20(9):e0331966. doi: 10.1371/journal.pone.0331966 (PMC12431195; doi:10.1371/journal.pone.0331966)
Supplement: Attachment — Submitted filename: #3.docx [file pone.0331966.s007.docx]

Dear Reviewer #3,

Thank you very much for reviewing my manuscript and for your positive evaluation.

In response to your comment regarding the resolution of the figures, I have improved their quality accordingly.

Thank you again for your valuable feedback.

Sincerely,
Tatsuya Koga
